# Hypercapnia Reduces Perceived Heat Pain in Healthy Subjects

**DOI:** 10.1002/ejp.70001

**Published:** 2025-02-13

**Authors:** A. Guekos, M. Hau, S. Grob, G. Sharvit, P. Schweinhardt

**Affiliations:** ^1^ Integrative Spinal Research, Department of Chiropractic Medicine, Balgrist University Hospital University of Zurich Zurich Switzerland; ^2^ Decision Neuroscience Lab, Department of Health Sciences and Technology, Institute of Human Movement Sciences and Sport ETH Zurich Zurich Switzerland; ^3^ Spital Limmattal Schlieren Switzerland; ^4^ iHomeLab Lucerne University of Applied Sciences and Arts Horw Switzerland; ^5^ Faculty of Medicine University of Zurich Zurich Switzerland

## Abstract

**Background:**

Danger signals modulate pain perception. Both amplification and attenuation of perceived pain are observed in healthy subjects exposed to danger signals, such as transient threats of an imminent electrical shock. However, exposure to danger signals in real life typically is not transient but constant over minutes to hours. Here, this was experimentally achieved by administering hypercapnic air (7.5% CO_2_). The primary objective was to investigate whether perceived heat pain would be differentially modulated during this intervention compared to regular air administration. The secondary objective assessed the potential differences of such a modulation with respect to heat intensity level.

**Methods:**

Thirty‐eight participants (19 women) received two air mixtures (hypercapnic and regular air) for 13 min each, during which 18 (6 × 3) noxious heat stimuli of three different intensities were applied to the calf and rated on two scales (intensity and pleasantness/unpleasantness). Psychological and physiological states were compared between conditions using the body sensations questionnaire, self‐assessment manikins, heart rate, and galvanic skin response. Statistical analyses were performed using Bayesian estimation testing.

**Results:**

Between‐condition differences were statistically meaningful for all heat intensity levels, always showing reduced pain perception during *hypercapnia* compared to *normocapnia*. The magnitude of the observed hypoalgesia did not depend on heat intensity levels.

**Conclusions:**

The presence of a continuous physiological danger signal results in hypoalgesia. Future studies need to determine whether the present results only hold for hypercapnia in healthy subjects or are generalisable to interactions between pain perception and continuous physiological danger signals in clinical pain populations.

**Significance Statement:**

It was shown that hypercapnia leads to reduced perception of noxious heat stimuli. If confirmed by neural data in future studies this could help to better understand the interaction of pain perception and continuous physiological danger signals in clinical pain conditions, potentially allowing for improved treatment of affected individuals.

## 1 Introduction

The presence of a danger signal, tangible, such as a burning fire, or abstract, such as an upcoming punishment, can modulate the perception of concurrent pain (Donnelly et al. 2020; Brandtstädter et al. 2004). This modulation follows transient changes in psychological state, such as fear or anxiety, that reflect the immediacy of the threat associated with the danger signal (Steimer 2002; Mobbs et al. 2009).

Depending on the state, opposite pain‐modulatory effects of danger signals are observed. Pain intensity is reduced by fear and augmented by anxiety (Rhudy and Meagher 2000). Such states are induced to study mechanisms of emotional changes involved in the maintenance and chronification of pain (Asmundson 2014; Ploghaus et al. 2001; Wiech and Tracey 2009; Frumkin and Rodebaugh 2021). However, contradictory results have sometimes questioned the respective induction paradigms (Rhudy and Meagher 2003).

Typically, phasic cues are used as danger signals, for example, threat of shock, presumably resulting in a gradually waning psychological state (Beaurenaut et al. 2020). However, danger signals in real life are often not phasic but sustained. To naturalistically test the signal's influence on the perception of concurrent pain, it should be continuously present. To that end, continuous physiological interventions are used and ideally measured over the whole experiment (Beaurenaut et al. 2020).

One well‐established physiological approach to continuously uphold a danger signal has subjects breathe hypercapnic air [7.5% carbondioxide (CO_2_), 21% oxygen (O_2_), 71.5% nitrogen (N_2_)]. This mimics a physiological danger signal by causing persistent shortness of breath (dyspnoea) during administration (Berliner et al. 2016). This paradigm is often used in behavioural studies of state anxiety (Woods et al. 1988; Bailey et al. 2005, 2007, 2011).

In contrast, it is rarely used with pain except for general dyspnoea paradigms that typically induce varying levels of hypercapnia, potentially associated with occasionally observed hypoalgesia (Morélot‐Panzini et al. 2007, Nishino et al. 1999, 2008). Only two studies examined the effects of fixed hypercapnia on pain perception. They reported increased thermal pain thresholds during the administration of 7.5% or 5% and 8% CO_2_ in O_2_, that is, combined hypercapnia and hyperoxia (Stokes et al. 1948; Grönroos and Pertovaara 1994). Given their age and possible confounding influences of hyperoxia (Abd‐Elsayed and Smith 2024), this potential pain modulation merits an updated investigation.

The present study's aim was to examine the effect of a physiological danger signal induced by continuous hypercapnia (7.5% CO_2_) without concomitant hyperoxia on pain perception in comparison to a neutral condition (regular air). Noxious heat stimuli were applied to the calf and rated for intensity and pleasantness/unpleasantness. Three intensities above the pain threshold were used with pain modulation expected to decrease with increasing intensity because higher intensity signals greater risk of physical harm that becomes more crucial to avoid (Morrison et al. 2013). Thus, the danger signal's relative importance and its effect on pain perception should decrease.

The primary objective was to test whether pain perception would differ between conditions. The secondary objective was to test whether such a potential modulation would be different according to applied stimulus intensity level.

## 2 Methods

### 2.1 General Study Design

The study followed a within‐subject experimental design with two consecutive conditions, called *normocapnia* and *hypercapnia*, during which participants were administered two breathable air mixtures through an oronasal face mask for a duration of about 13 min each. For *normocapnia*, the mixture corresponded to regular (i.e., normocapnic) room air. For *hypercapnia*, the CO_2_ concentration was increased to 7.5% (i.e., hypercapnic air). In both conditions, 18 noxious heat stimuli of three different suprathreshold intensities were applied in randomised order (identical for both conditions) to three locations on the skin over the medial calf of either the left or the right leg. Every stimulus had to be rated on two visual analogue scales. The order of conditions and the choice of testing leg were counterbalanced across participants.

Before the experiment and at the end of both conditions, participants had to fill in the Body Sensations Questionnaire (BSQ) consisting of 17 items relating to the perception of bodily sensations rated on a five‐point Likert scale, which was originally developed to assess how persons suffering from agoraphobia respond to perceived changes in bodily sensations (Chambless et al. 1984). It can also be used to assess panic‐specific anxiety sensitivity (Ehlers and Margraf 1993; Richards et al. 2001; Ohst and Tuschen‐Caffier 2020a, 2020b; Zahler et al. 2020). Here, it primarily served as a psychological manipulation check, that is, to test whether participants were conscious of the physiological intervention, that is, perceiving changes in their bodily sensations between *hypercapnia* and *normocapnia*. Additionally, valence and arousal ratings were obtained using the self‐assessment manikins (SAM) (Bradley and Lang 1994) at the beginning, in the middle, and at the end of each condition. Ratings were compared between conditions, and potential changes in affective reaction over time within conditions were monitored.

Study participation consisted of one experimental session of 2 h, including screening and familiarisation with the testing procedures. Participants were pseudorandomly assigned a session time in the morning or in the afternoon to reduce the influence of daytime on the results (Labrecque and Vanier 1995). Anthropometric data were collected right before testing. Written informed consent was obtained from all participants.

The study was approved by the Ethical Board of the Canton of Zurich (Kantonale Ethikkomission Zürich) and registered at kofam.ch (trial number SNCTP000004458). The study observed the principles of the declaration of Helsinki.

### 2.2 Participants

The study cohort included men and women in good general health and with a body‐mass index (BMI) between 18 and 31. The age range of participants was limited to 18–40 years to reduce influences of age on study results. In order to minimise the potential effects of learned coping strategies from prior experiences, study participation was restricted to persons with no history of repeated purposeful or accidental exposure to non‐normocapnic breathing. For instance, divers, high‐altitude climbers, endurance athletes exercising more than twice per week, or individuals suffering from acute or chronic respiratory diseases were ineligible for study participation.

Analysis of pilot measurements for the experimental design allowed us to determine the necessary sample size of 36 using a two‐sided *t* test with an effect size of 0.45, a significance level of 0.05 and a power of 0.75. To account for potentially unusable data, it was decided to include 38 participants. Respecting the Sex and Gender Equity in Research (SAGER) guidelines (Heidari et al. 2016), it was aimed at recruiting an equal number of men and women, although no sex‐specific differences were expected from the pilot data analysis.

#### 2.2.1 Exclusion Criteria

General exclusion criteria were any major medical or psychiatric conditions, any chronic pain conditions, back pain for more than three consecutive days over the past 3 months, intake of any analgesics, or consumption of any intoxicants in the past 48 h before the experiment, scar tissue or known reduced sensitivity on the calf.

Exclusion criteria relating to the administration of hypercapnia were any cardiovascular, neurological, or thyroid disorders, any personal or family history of anxiety attacks or disorders, any personal or family history of panic attacks or disorders, any history of epilepsy or seizures, any primary or secondary headache diseases, any sleeping disorders, high caffeine or alcohol consumption, and any past or present smoking habits.

If a participant could not endure the air mixtures or the thermal protocol, the experiment was terminated, and the participant was excluded and replaced with respect to sample size.

Because the present study investigated potential modulatory effects of hypercapnia on the perception of different suprathreshold heat stimuli, data from participants who could not distinguish between the three intensities of the stimuli during *normocapnia* were excluded from the study cohort and not analysed. The respective participants were replaced with respect to sample size.

### 2.3 Experimental Setup

During familiarisation and testing, participants sat in a semi‐supine position on a test bed. The backrest was inclined at 70°–80° to facilitate comfortable viewing of the computer screen where instructions and feedback prompts were displayed. The screen was placed on a hospital‐bed table in front of the participant at a distance of 40–50 cm together with a response mouse. Underneath the table, the lower legs lay relaxed on a wedge‐shaped foam pillow to ensure easy access to the medial calf, where three locations were marked along the cranial‐caudal axis.

Over the whole experiment, autonomic recordings from the participant were acquired using a PowerLab 8/35 (ADInstruments, Dunedin, NZ) biological data acquisition system at a 1 kHz sampling frequency. They included three‐point electrocardiography (ECG; range: ±100 mV; FE231 Bio Amp, ADInstruments) from the upper torso using shielded lead wires (98 cm, MLA2503, ADInstruments) and stick‐on disposable electrodes (MLA1010B, ADInstrument), galvanic skin response (GSR; range: ±40μS at 1 V to 20 μS conversion; FE116 GSR Amp, ADInstruments) from the foot (contralateral to the testing side) using bipolar electrodes (3 × 2.5 cm, MLT118F, ADInstruments) at the big and middle toes and oxygen/carbon dioxide concentrations (ML206 Gas Analyzer, ADInstruments). One of the two experimenters continuously monitored these data to ensure participant safety and compliance with the experimental protocol. ECG and GSR recordings were used for physiological manipulation checks (see Section 2.6).

For delivery of the air mixtures over the course of the experiment, the participant was fitted with a single‐use oronasal face mask and a two‐way breathing circuit assembly, details of which have previously been published (Tancredi et al. 2014).

For *normocapnia*, the air mixture corresponded to regular room air, that is 21% O_2_, 0.04% CO_2_ and the rest N_2_. For *hypercapnia*, the composition was 21% O_2_, 7.5% CO_2_, and the rest N_2_, that is, hypercapnic air with the same oxygen content as in regular air. The two air mixtures were ordered as medicinal gas (PanGas, Dagmersellen, CH) in pressurised bottles of 10 L volume under 200 bar (Linde plc, Dublin, IE).

About half an hour before the experiment, the two mixtures were filled into four separate Douglas bags (two per mixture) of 120 L volume each (ADInstruments, Dunedin, NZ). Upon filling, the mixtures were heated and humidified using a medical humidification device (MR810, Fisher & Paykel, East Tamaki, NZ) to reach typical ambient room air conditions, that is, approximately 20°C and 60% humidity.

The four bags were mounted onto a customised, in‐house‐built wooden frame, the so‐called *hypercapnibar* (height 125 cm, width 152 cm, depth 11.5 cm, plank thickness 3 cm), which had three 3‐way directional manual stopcocks with a 2.86 cm inner port diameter (ADInstruments, Dunedin, NZ) screwed onto the top plank, one in the centre and one at each end. The side ports of the central stopcock were connected by 62 cm long plastic tubes (Fisher & Paykel, East Tamaki, NZ) to the middle ports of the outer stopcocks. The Douglas bags were attached to the side ports of the outer stopcocks, one with two bags of regular air and one with two bags of hypercapnic air. The two outer stopcocks were always open between the bags containing the same mixture. By switching the flow direction of the central port, the experimenter was thus able to choose the mixture to be delivered to the participant.

**FIGURE 1:**
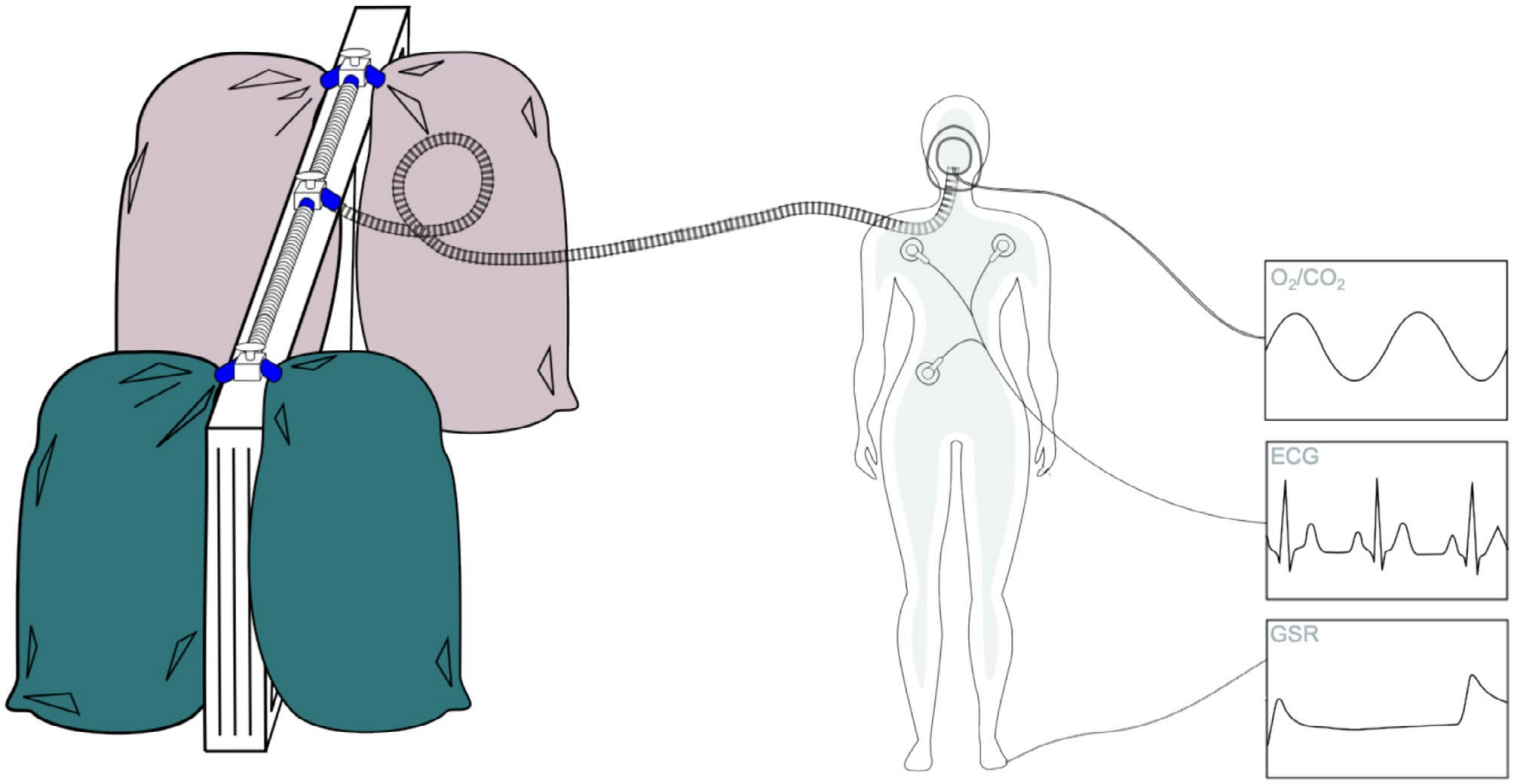
Schematic of the participant setup. Left side: In‐house‐built wooden frame (*hypercapnibar*) with four Douglas bags, two for each mixture (normocapnic and hypercapnic) indicated by different colours (dark green and light brown). Bags of the same mixture are connected through three‐way directional manual stopcocks, which are themselves connected by tubes to a central stopcock, from which another tube delivers the mixture to the oronasal face mask of the participant. Right side: Participant representation with three measuring sites of autonomic recordings. Oronasal face mask with sampling tube to monitor oxygen (O_2_) and carbondioxide (CO_2_), three electrodes on the torso (distally below left and below right clavicule and distally above right hip) for electrocardiography (ECG), one pair of electrodes at the left foot (attached to the big and middle toes) for recording galvanic skin response (GSR).

From the central stopcock, the mixtures were delivered via a 250 cm long plastic tube (Teleflex Inc., Wayne, US) to the participant. A schematic of the *hypercapnibar* integrated into the participant setup is shown in Figure 1.

Noxious heat stimuli were delivered using a 3 cm × 3 cm computer‐controlled peltier thermode (Pathway Pain & Sensory Evolution System, Medoc, St. Ramat Yishai, IL). The thermode was manually moved by the second experimenter from one location on the calf to the next after every stimulus to avoid potential habituation or sensitisation to the heat stimuli.

### 2.4 Familiarisation

Participants were familiarised with the air mixtures and the noxious heat stimuli. The order of familiarisation was counterbalanced across participants. For the air mixtures, the familiarisation consisted of a consecutive administration of the mixtures for 2 min each. The starting mixture was counterbalanced across participants. Beforehand, they were informed of the respective compositions of the mixtures but not of any potential physiological or psychological reactions to them. They were told that both mixtures contained the same concentration of O_2_, the physiologically relevant component in air, but different concentrations of CO_2_, with one corresponding to regular air and the other to 7.5% CO_2_ hypercapnic air. Furthermore, they were informed that they might notice a difference while breathing one or the other, which would be perfectly normal. For the heat stimuli, every participant received a continuously increasing heat stimulus starting at a baseline of 32°C and ramping up at 1°C/s until the participant pressed a button on a computer mouse. This procedure was performed twice on two adjacent locations of the calf (contralateral to the subsequent testing side) to determine the individual heat pain and heat tolerance thresholds, respectively defined as 100 and 200 on a scale from 0 (no sensation) to 200 (most intense pain tolerable) (Villemure et al. 2003; Becker et al. 2013).

To ensure familiarity with the scale, the familiarisation procedures were followed by the application of four tonic heat stimuli of 6 s duration (ramp up/down of 10°C/s from 32°C baseline) to the same calf using all three pre‐marked locations. The respective temperatures, applied in random order, were calculated based on the two obtained individual thresholds to theoretically correspond to ratings between 120 and 190, relying on the observation of linearly increasing heat pain perception (Lautenbacher et al. 1992). Between stimuli, participants were given 30 s for verbal intensity rating of the perceived mean sensation. In case of inability to discriminate the applied stimuli from one another, the procedure was repeated a maximum of twice. If no improvement was observed, the participant was dismissed and excluded.

### 2.5 Experimental Protocol

Right before the start of the experiment, the heat pain and heat tolerance thresholds were determined thrice, that is, once at each of the three locations of the test leg, using the same procedure as during the familiarisation. Individual results of the three measurements were noted and their means calculated.

**FIGURE 2:**
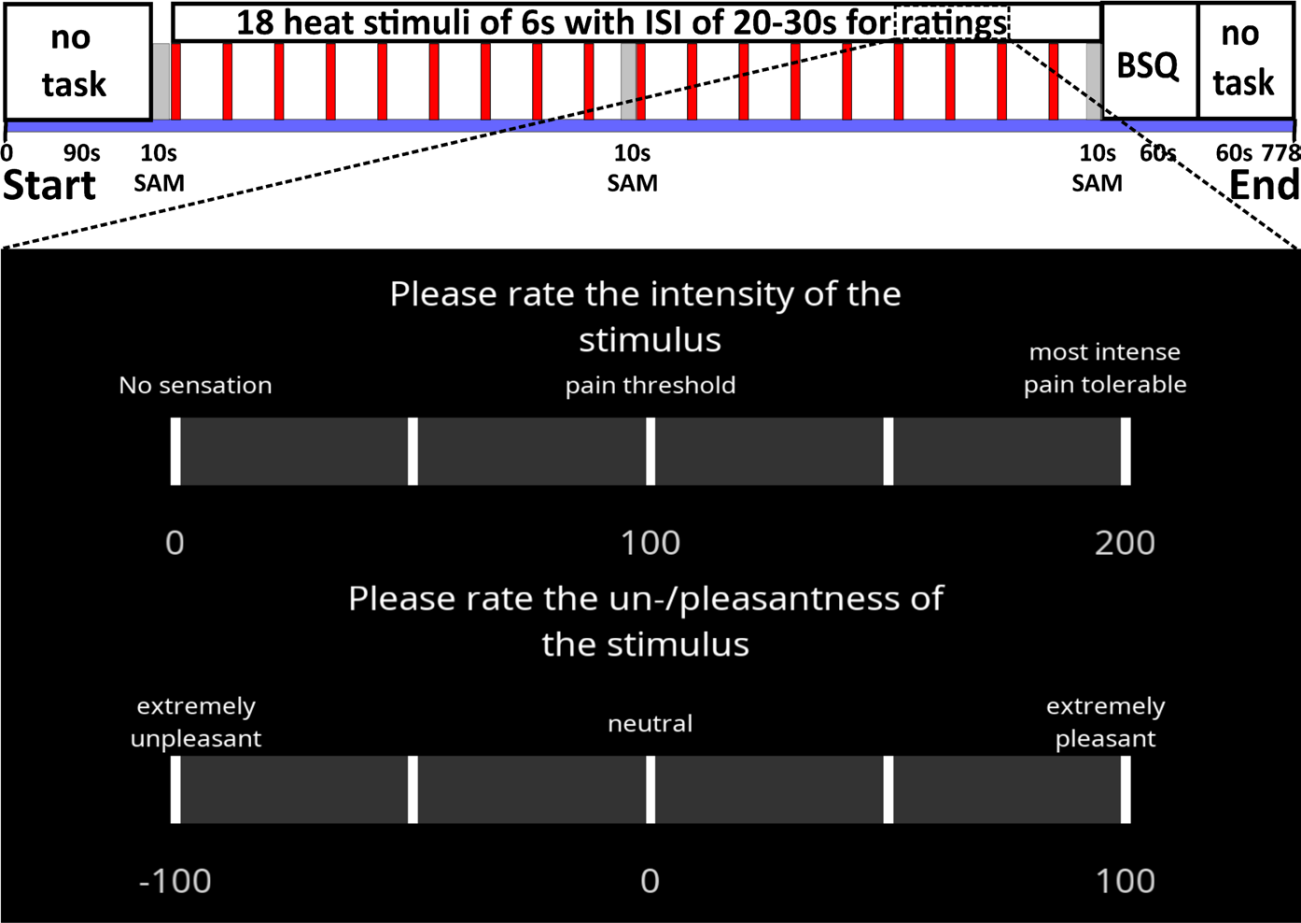
Experimental protocol per condition. Top: Time course of the experiment starting with 90 s of task‐free time followed by 10 s for valence and arousal ratings on self‐assessment manikins (SAM). Eighteen heat stimuli of 6 s duration are applied with interstimulus intervals (ISI) of 20–30 s (mean = 25 s) for ratings. SAM are again displayed in the middle and after the last stimulus ratings. The Body Sensations Questionnaire (BSQ) is displayed for 60 s, followed by 60 s of task‐free time. Bottom: The two rating scales for the heat stimuli as displayed during every ISI for 15 s.

The two conditions, *normocapnia* and *hypercapnia*, were identically structured (Figure 2). Each condition began with a task‐free phase of 90 s during which the participant just had to breathe the respective air mixture. Then, the first valence and arousal ratings (10 s) had to be completed by clicking with the mouse cursor on the respective SAM icons displayed on the computer screen before the application of the noxious heat stimuli started. SAM ratings were prompted again in the middle of the condition, that is, during the ninth interstimulus interval and after rating the last, that is, the 18th stimulus. Subsequently, the participant was given 60 s to reply to the displayed BSQ. Participants were informed that the BSQ would be presented after the last heat stimulus of each condition in order to avoid a potential influence of heat stimulus anticipation on the BSQ score. After a further 60 s of task‐free time, the air mixture was switched, and the entire procedure was repeated for the second condition.

The 18 phasic heat stimuli consisted of three times six stimuli of three different intensity levels, termed low, medium, and high. Following extensive pilot testing, the respective temperatures were calculated for each participant based on the individual heat tolerance threshold, with low intensity corresponding to 95%, medium to 96.5%, and high to 98% of the tolerance threshold in order to apply three well‐distinguishable intensities. The stimuli were applied in randomised order in the first condition, which was kept identical for the second condition. Each stimulus was applied to one of the three pre‐marked locations on the medial calf in alternating order for a duration of 6 s (ramp up/down of 10°C/s from 32°C baseline), which was directly followed by a rating period of 15 s. The total interstimulus interval was set to randomly vary between 20 and 30 s (mean = 25 s). Alternating between application locations was performed by one of the experimenters who manually shifted the thermode between stimuli.

The participants rated the perceived intensity of each stimulus from 0 to 200 during familiarisation and, additionally, the perceived pleasantness/unpleasantness. The pleasantness/unpleasantness scale ranged from −100 (extremely unplesant) to 100 (extremely pleasant) with 0 corresponding to neutral (Villemure et al. 2003; Becker et al. 2013). Both scales were presented as horizontal bars with anchors for the lowest, highest, quarter‐, and mid‐point values. Ratings had to be filled in using a mouse‐controlled slider on the two scales displayed on the screen (Figure 2). The whole protocol was programmed and displayed with PsychoPy v3.2021.2.3 (Open Science Tools Ltd., Nottingham, UK) (Peirce et al. 2019). The total duration of each condition was approximately 13 min (778 s).

Right after the experiments, participants were additionally asked to describe in their own words how they felt during the two conditions.

### 2.6 Data Analysis

To test whether the two conditions were successfully implemented and could be distinguished, four manipulation checks were included: two psychological and two physiological ones. First, the BSQ score was compared between conditions and to the baseline (score before the experiment). Second, SAM scores for valence and arousal were compared between conditions for each of the three measurement time points. Third and fourth, mean heart rate (HR), calculated from ECG recordings as beats per minute (bpm) and mean GSR, given as change in μS from individual baseline (set to 0) before the experiment, were compared between conditions.

The study's primary objective was to test for an influence of the *hypercapnia* condition on perceived pain compared to the *normocapnia* condition. It was therefore assessed whether differences in perceived intensity or pleasantness/unpleasantness ratings between the two conditions were statistically meaningful. To address the secondary objective, whether potential modulatory effects of *hypercapnia* were more pronounced depending on the intensity level of the applied stimuli, the differences between conditions were also compared across the three levels (low vs. medium, low vs. high and medium vs. high).

As exploratory analyses, correlations between changes in pain ratings (intensity and pleasantness/unpleasantness) between conditions and changes in any of the four metrics used for manipulation checks (BSQ, SAM, HR and GSR) between conditions were examined across participants. Furthermore, SAM scores were tested within the condition for potential changes over time.

All experimental data were stored per participant in PsychoPy as comma‐separated value files (CSV), from which they were extracted and saved together with the anthropometric data in a combined CSV. Instances of missing experimental data due to slow reaction by the participant were not replaced nor imputed. This concerned 0 intensity and 1 (0.07%) unpleasantness ratings as well as 25 (5.48%) SAM and 32 (1.65%) BSQ responses. For the BSQ, the analyses were performed using normalised scores, that is, total scores divided by the number of answered questions.

Data analysis was done in R 4.2.3 (R Foundation for Statistical Computing, Vienna, Austria) with ‘tidyverse’ collection of packages and the packages coda 0.19‐4, BEST 0.5.4, rjags 4.13 and runjags 2.2.1‐7 relying on JAGS 4.3.0. Statistical testing was carried out in a Bayesian framework observing the recommended reporting guidelines (Kruschke 2021) whenever applicable. Statistical comparisons relied on Bayesian estimation testing using the BEST package in R for execution in JAGS. Bayesian estimation testing is a very robust method to determine differences between groups. It does not require specific assumptions with respect to data distribution or outliers as do more traditional tests, such as the student's *t* test (Kruschke 2013). It is ideally suited for behavioural studies that have an upper limit on the number of potential repetitions of interventions, as is often the case in pain studies, because the method allows for straightforward interpretation of the performed analysis without requiring a high number of data points to do so (Dunson 2001).

For Bayesian estimation testing, broad uninformed priors were used. Calculations of the posterior distributions were done with three sampling chains with 10,000 iterations, a burn‐in of 3000 and a thinning factor of 3. Sampling chains were considered to converge well if the Brooks‐Gelman‐Rubin scale reduction factor lay below 1.05 (Gelman and Rubin 1992).

Statistical results are reported with the 95% highest density interval (HDI_95_). If the HDI_95_ included the value 0, that is, ‘no difference’, the compared differences were considered not statistically meaningful. Effect sizes were calculated as Cohen's *d* (Cohen 1988; Sawilowsky 2009) whereby the mode of the respective HDI_95_ is reported.

## 3 Results

Sixty‐one subjects (28 men and 33 women) were recruited through online advertisement on a dedicated platform of the University of Zurich as well as by word of mouth. Three subjects were excluded during screening because of previously undetected exclusion criteria: past or present smoking habits and chronic pain, respectively. Ten participants (3 men and 7 women) aborted the experiment during familiarisation (9) or *hypercapnia* (1) because of not tolerating the hypercapnic air mixture. Data from two participants were unusable due to technical problems (device/equipment failure). Eight participants were unable to distinguish between the three intensity levels of the applied heat stimuli. They were excluded and replaced with respect to sample size. In total, data from 38 included participants (19 men and 19 women) aged 20–37 years (24.7 ± 3.9) and with a BMI between 18 and 31 kg/m^2^ (22.8 ± 3.3) were analysed.

### 3.1 Study Cohort

Normalised BSQ scores before the start of the experiment ranged from 0 to 1.76 (0.20 ± 0.38) indicating no elevated panic‐specific anxiety sensitivity at baseline (Ohst and Tuschen‐Caffier 2020a, 2020b; Zahler et al. 2020). Table 1 shows the characteristics of the study cohort, including the obtained heat pain and heat tolerance thresholds on the test side. Following SAGER guidelines, these characteristics were compared between men and women. Men were slightly older than women (3.2 ± 5.6 years). Differences in height, weight, and BMI were statistically meaningful, as was to be expected from the distribution in the general population. Importantly, neither BSQ total scores nor heat pain or heat tolerance thresholds differed between the sexes. This confirmed observations in pilot experiments. Furthermore, studies using similar exposures to hypercapnic air mixtures have also not reported sex‐specific differences, to the best of the authors' knowledge. Thus, the present experimental data was not separately analysed for men and women.

**TABLE 1:** Characteristics of the study cohort (*n* = 38 [19 men and 19 women]).

	Full Cohort (mean ± SD)	Men (mean ± SD)	Women (mean ± SD)	HDI_95_ (men‐women)
Age [years]	24.7 ± 3.9	26.3 ± 4.5	23.1 ± 2.3	**[0.7, 5.6]**
Height [cm]	172.6 ± 9.5	178.8 ± 5.9	166.3 ± 8.1	**[8.2, 18.0]**
Weight [kg]	68.3 ± 13.6	76.9 ± 10.5	59.8 ± 11.4	**[10.1, 25.3]**
BMI [kg/m^2^]	22.8 ± 3.3	24.0 ± 2.9	21.5 ± 3.2	**[0.7, 4.8]**
BSQ (normalised total score)	0.20 ± 0.38	0.19 ± 0.28	0.22 ± 0.46	[−0.04, 0.14]
Heat pain threshold [°C]	42.5 ± 1.9	42.1 ± 2.2	42.8 ± 1.5	[−2.1, 0.5]
Heat tolerance threshold [°C]	50.3 ± 1.7	50.8 ± 1.9	49.9 ± 1.5	[−0.1, 2.1]

*Note:* Bold HDI_95_ values indicate statistically meaningful differences.

Abbreviations: BMI, body‐mass index; BSQ, Body Sensations Questionnaire before the experiment (i.e., at baseline). The outermost column shows the HDI_95_ for the difference in means (men‐women) after Bayesian estimation testing; cm, centimetres; HDI_95_, 95% highest density interval for difference in means (men‐women) of posterior distribution after Bayesian estimation testing; SD, standard deviation.

### 3.2 Manipulation Checks

Normalised BSQ scores rose from 0.20 ± 0.38 at baseline (before the experiment) to 0.73 ± 0.43 during *hypercapnia* and to 0.34 ± 0.30 during *normocapnia*. The difference between the two conditions was statistically meaningful (HDI_95_ = [0.21, 0.47], Cohen's *d* = 1.08). The difference to baseline was meaningful as well, for *hypercapnia* (HDI_95_ = [0.38, 0.67], Cohen's *d* = 1.23) as expected but also – to a lesser extent –for *normocapnia* (HDI_95_ = [0.06, 0.24], Cohen's *d* = 0.58).

At all three measurement time points, valence and arousal ratings statistically differed between conditions. For arousal, ratings were higher during *hypercapnia* compared to *normocapnia* with mean differences of 0.76 (HDI_95_ = [0.16, 1.34], Cohen's *d* = 0.87) at beginning, 1.06 (HDI_95_ = [0.54, 1.62], Cohen's *d* = 1.99) in the middle, and 1.00 (HDI_95_ = [0.83, 1.18], Cohen's *d* = 4.63). Valence ratings were lower at all three time points with mean differences of −0.63 (HDI_95_ = [−1.19, 0.10], Cohen's *d* = 0.79), −0.70 (HDI_95_ = [−1.18, −0.21], Cohen's *d* = 1.00) and − 0.58 (HDI_9_5 = [−1.02, −0.14], Cohen's *d* = 0.94), respectively. Within condition, valence and arousal ratings remained stable over time (data not shown).

Mean HR differed between *normocapnia* (71.24 ± 9.61 bpm) and *hypercapnia* (75.61 ± 10.07 bpm). The difference was statistically meaningful (HDI_95_ = [2.22, 6.11], Cohen's *d* = 0.75). Similarly, mean GSR was higher during *hypercapnia* (3.79 ± 4.53 μS) compared to *normocapnia* (2.56 ± 4.10 μS). The difference was statistically meaningful (HDI_95_ = [0.31, 1.83], Cohen's *d* = 0.50).

All participants reported feeling uncomfortable during *hypercapnia* due to perceived breathing difficulties. About 34% (13 of 38) described feeling anxious or close to panicking. There was no difference between these participants and the rest with respect to manipulation checks nor stimulus ratings (data not shown).

### 3.3 Reduction of Perceived Pain During Hypercapnia

**FIGURE 3:**
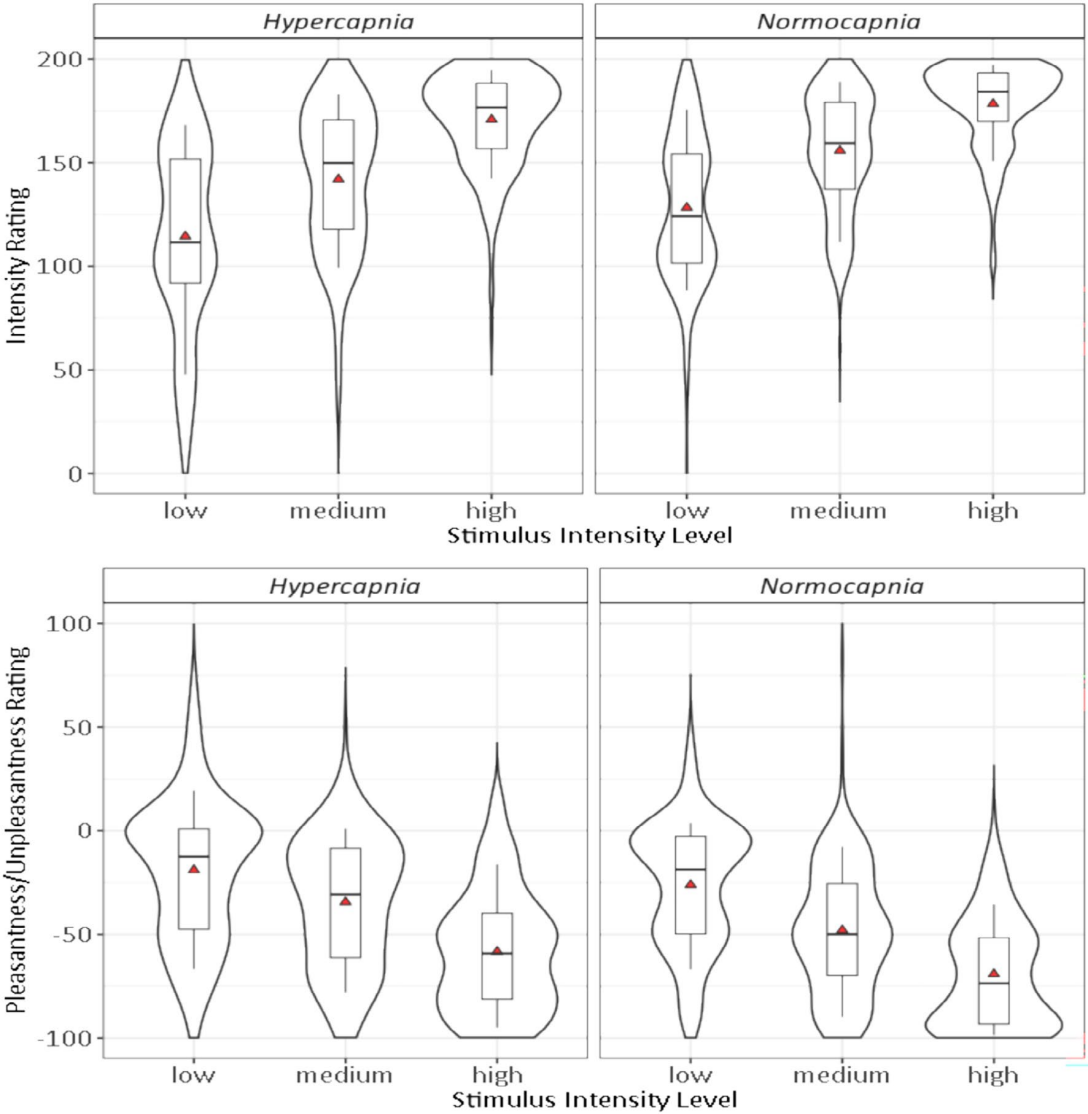
Ratings of heat stimuli by condition (*hypercapnia* and *normocapnia*) w.r.t. stimulus intensity level (low, medium and high). Top: Intensity ratings from 0 (no sensation) to 200 (most intense pain tolerable) with 100 = pain threshold. Bottom: Pleasantness/unpleasantness ratings from −100 (extremely unpleasant) to 100 (extremely pleasant) with 0 = neutral. Violinplots delineating the distribution of the data with boxplots indicating the 25%–75% interquartile range and median (thick horizontal line), with whiskers extending to 10% and 90%, respectively, and means given as red triangles.

Intensity and unpleasantness were reduced during *hypercapnia* compared to *normocapnia* (Figure 3). Participants rated the heat stimuli lower on the intensity scale, that is, less painful. This was the case for all three stimulus intensity levels, that is, for low (114.5 ± 44.4 vs. 128.3 ± 34.6), medium (142.0 ± 34.8 vs. 155.8 ± 28.3) and high (170.8 ± 23.0 vs. 178.4 ± 20.0). Similarly, the stimuli were rated higher on the pleasantness/unpleasantness scale, that is, less unpleasant for low (−19.0 ± 34.4 vs. −26.1 ± 30.2), medium (−34.4 ± 31.6 vs. −48.0 ± 30.5) and high (−58.4 ± 28.6 vs. −69.2 ± 25.8). For both rating types, the differences between *normocapnia* and *hypercapnia* were statistically meaningful overall as well as for every stimulus intensity level separately (Table 2) with effect sizes from 0.40 to 0.43 for differences in intensity ratings and from 0.33 to 0.42 for differences in pleasantness/unpleasantness ratings.

**TABLE 2:** Difference in ratings [HDI_95_] (effect size) between conditions (*normocapnia‐hypercapnia*) w.r.t. stimulus intensity levels.

	All levels	Low	Medium	High
Intensity	**[7.8, 12.0] (0.42)**	**[7.8, 16.2] (0.43)**	**[8.9, 17.4] (0.43)**	**[4.7, 10.3] (0.40)**
**Pleasantness/unpleasantness**	**[−10.1, −6.2] (0.38)**	**[−8.8, −3.1] (0.33)**	**[−16.2, −7.5] (0.42)**	**[−11.8, −5.4] (0.42)**

*Note:* Effect size is given in brackets () as the mode of the distribution for Cohen's *d*. Bold HDI_95_ values indicate statistically meaningful differences. HDI_95_: 95% highest density interval of posterior distribution after Bayesian estimation testing; Intensity: difference in intensity rating on a 0–200 scale with 0 = no sensation, 100 = pain threshold and 200 = most intense pain tolerable; Pleasantness/Unpleasantness: difference in pleasantness/unpleasantness rating on a −100 to 100 scale with −100 = extremely unpleasant, 0 = neutral and 100 = extremely pleasant; Low/medium/high: stimulus intensity level.

### 3.4 No Intensity‐Level Dependent Modulation

Between‐level comparison of differences in intensity ratings and in pleasantness/unpleasantness ratings revealed no statistically meaningful differences (Table 3). Thus, the observed modulatory effect did not statistically differ with respect to stimulus intensity level.

**TABLE 3:** Pairwise comparison of ratings' differences (*normocapnia*‐*hypercanpia*) [HDI_95_] (effect size) between levels.

	Low vs. medium	Low vs. high	Medium vs. high
Intensity	[−6.3, 5.3] (0.02)	[−0.7, 9.1] (0.18)	[−0.1, 10.0] (0.21)
Pleasantness/unpleasantness	[−0.4, 9.4] (0.20)	[−1.6, 6.8] (0.13)	[−6.9, 3.2] (0.07)

*Note:* Effect size is given in brackets () as the mode of the distribution for Cohen's *d*. HDI_95_: 95% highest density interval of posterior distribution after Bayesian estimation testing; Intensity: difference of differences in intensity rating on a 0–200 scale with 0 = no sensation, 100 = pain threshold, and 200 = most intense pain tolerable; Pleasantness/Unpleasantness: difference of differences in pleasantness/unpleasantness rating on a −100–100 scale with −100 = extremely unpleasant, 0 = neutral, and 100 = extremely pleasant; low/medium/high: stimulus intensity level.

### 3.5 No Correlation Between Observed Pain Modulation and Manipulation Read‐Outs

The changes between conditions in perceived pain (intensity and pleasantness/unpleasantness) did not correlate for any of the three stimulus intensity levels with the changes in the four measures (BSQ, SAM, HR and GSR) used to confirm the physiological manipulation (data not shown).

## 4 Discussion

The present study observed that noxious heat stimuli were perceived as less painful and less unpleasant during *hypercapnia* compared to *normocapnia*. This observed modulation did not differ with respect to stimulus intensity level. Manipulation checks using BSQ and SAM (arousal and valence dimensions) scores as well as HR and GSR indicated the continuous presence of a physiological danger signal during *hypercapnia*.

### 4.1 Influence of Stressor Characteristics

The present hypercapnic challenge (7.5% CO_2_ over 13 min) represents an external stressor that disrupts bodily homeostasis and causes adaptive physiological and psychological reactions, which were reflected in three manipulation checks (HR, GSR and SAM). As an experimental manipulation, the hypercapnic challenge has three characteristics: it is uncontrollable, unavoidable, and predictable, especially with respect to its onset and duration. All three characteristics have been shown to influence sensory perception.

First, exposure to an uncontrollable physiological stressor has been shown to affect measures of emotional and physiological state, such as anxiety or GSR (Breier et al. 1987; Maier and Watkins 2005; Havranek et al. 2016). Accordingly, increases in such measures are typically reported for the hypercapnic challenge used in the present study (Woods et al. 1988; Bailey et al. 2005). In turn, these increases have been related to pain‐modulatory effects, such as hyperalgesia following anxiety induction by phasic danger signal cues (Cornwall and Donderi 1988; Rhudy and Meagher 2000). Nevertheless, uncontrollability of a danger signal has also been observed to decrease perceptual sensitivity to concomitant painful stimuli (Brandtstädter et al. 2004), potentially because more attention is directed away from the stimuli by certain experimental paradigms than by others (Rhudy and Meagher 2003; see Section 4.2). Second, if the stressor is perceived as unavoidable, subjective reports of anxiety and GSR have been demonstrated to increase (Beaurenaut et al. 2020). Such an increase has typically been associated with hyperalgesia (Rhudy and Meagher 2000). Third, predictability seems connected to increased context processing, that is, enhanced neural activity associated with the stressor (Wieser et al. 2016), while unpredictability of a stressor leads to increased vigilance (Matthews et al. 1980; Wieser et al. 2016). Thus, predictability of a stressor would shift attentional resources away from concomitant painful stimuli and potentially lead to hypoalgesia (McCaul and Malott 1984; see Section 4.2). Indeed, such a shift in attention has been demonstrated for the hypercapnic challenge used in the present study by selectively improved temporal detection and spatial discrimination of unrelated visual cues (Garner et al. 2011, 2012).

In sum, of the three characteristics, predictability would be expected to have a hypoalgesic effect, unavoidability a hyperalgesic one, and uncontrollability a hypo‐ or hyperalgesic one, depending on the details of the experimental paradigm. A hypoalgesic effect is linked to a shift in attention away from the painful stimuli, a hyperalgesic one to a potential increase in state anxiety or general vigilance. In the present study, the latter might be partially reflected in the elevated BSQ scores during hypercapnia compared to normocapnia, whereas an attentional shift was not visible in a change in reaction times during ratings (data not shown; see Section 4.2). However, the three discussed characteristics and their respective contributions to the outcome measures are dependent on each other. For instance, it has been shown that uncontrollability and unpredictability can interact with respect to measures of psychological state (Havranek et al. 2016).

Thus, the observed hypoalgesia should not be attributed to just one determining characteristic of the hypercapnic challenge. Furthermore, the paradigm's continuous nature, that is, the 13‐min long uninterrupted administration of hypercapnic air, might be decisive with respect to the overall binding of attentional resources and to understanding the observed hypoalgesia.

### 4.2 Hypercapnia Diverts Attention Away From Pain

Shifting attention away from a painful stimulus has a hypoalgesic effect (McCaul and Malott 1984), whereby the effect's magnitude may depend on individual subjective trait characteristics (Rischer et al. 2020; Asefi Rad and Wippert, 2024). The administered continuous hypercapnic challenge has been shown to not only divert attention (Garner et al. 2011, 2012) but also to negatively affect prefrontal executive function (Savulich et al. 2019), while not affecting goal‐directed behaviour (Gillan et al. 2021). The latter result would explain the absence of a difference in reaction times for pain ratings, while the former might be the principal reason for the ratings' decrease during *hypercapnia* compared to *normocapnia* and for the seeming discrepancy with other studies.

Previous studies involving danger signals have mostly observed hyperalgesia (Cornwall and Donderi 1988; Benedetti et al. 1997, 2006; Rhudy and Meagher 2000; Metzger et al. 2019). As pointed out before, the applied danger signals were not continuously present in any of these studies because the associated manipulation typically involved a transient external cue, such as a threat of shock. Intermittently present danger signals might not have the same attentional effect on pain perception because the activated mechanisms would already be waning when the painful stimuli are being processed. By consequence, a potential hypoalgesic effect due to the shift in attention would either already have disappeared upon pain perception measurement in a majority of subjects (Metzger et al. 2019). Or, the effect would have become hyperalgesic due to a renewed increase in attention to the painful stimuli, consistent with previous interpretations (Rhudy and Meagher 2000; Cornwall and Donderi 1988). Thus, the present results do not necessarily contradict previously published results.

### 4.3 Potential Neural Mechanisms

The reduction of perceived heat pain during *hypercapnia* is in line with the few available studies from clinical and pre‐clinical research assessing the influence of hypercapnia on pain perception (Stokes et al. 1948; Gamble and Milne 1990; Grönroos and Pertovaara 1994). Supposedly, the hypercapnia‐dependent increase in the firing rate of locus coeruleus (LC) neurons for concentrations above 5% CO_2_ (Elam et al. 1981; Pineda and Aghajanian 1997; Oyamada et al. 1998) resulted in a depression of nociceptive transmission at the dorsal horn (Gamble and Milne 1990), in line with observed LC‐dependent firing rate modulation (Mokha et al. 1985). However, LC firing is also triggered by acute pain and is considered a robust response to such an acute stressor, resulting in spinally mediated hypoalgesia (Suárez‐Pereira et al. 2022). There is evidence to suggest that the corresponding LC activity is regulated by endogenous opioid release during stress, whereby firing rates change from phasic in the initial stage to the tonic in the continuous stage, presumably to allow for behavioural and attentional adaptation (Valentino and Van Bockstaele 2015).

Depending on the experimental paradigm, phasic LC firing would be triggered following a brief transient stimulus, such as an acute painful stimulus or a threat, whereby the resulting hypoalgesic effect might be brief (see Section 4.3). But tonic LC firing would be present during sustained input, such as the present hypercapnic challenge, resulting in a longer‐lasting hypoalgesia. In sum, it could be speculated that the presently observed hypoalgesia during *hypercapnia* as well as the well‐documented hypoalgesia following acute stress both result from induced LC activity although the underlying neuronal firing patterns might differ.

### 4.4 No Differential Pain Modulation With Respect to Stimulus Intensity

The secondary result, that is, the absence of a differential modulation on perceived pain with respect to stimulus intensity level during *hypercapnia*, seems counterintuitive. The perception of high‐intensity stimuli should be less reduced compared to low or medium intensity ones. Yet, the present results suggest that a continuously present danger signal, such as *hypercapnia*, reduces the prioritisation of higher over lower intensity stimuli, potentially because the physiological danger signal binds the attentional resources (see Section 4.2) and impedes a nuanced assessment of the stimuli. High‐intensity stimuli become more innocuous than they would otherwise be perceived. Such an interference with the brain's natural defensive reactivity patterns could lead to partly ignoring the risk of potential physical harm in individuals suffering from continuous state anxiety over extended periods of time. They would thus be more likely to misjudge the relevance of peripheral nociceptive input.

### 4.5 Conclusions

Heat pain stimuli were perceived as less intense and less unpleasant during *hypercapnia* induced by the administration of hypercapnic air. The results contrast with the available literature reporting hyperalgesia following phasic cues for danger signals. It is hypothesized that the presence of a continuous danger signal activated other neural mechanisms than the phasic induction paradigms so far used in pain research. If future studies confirm differential mechanisms, this opens a new perspective to potentially better understand interactions between pain and unrelated danger in patients.

## Author Contributions

All authors contributed to the pilot measurements and literature research; A.G. and M.H. designed the study; A.G., M.H., and S.G. performed the experiments and data collection; A.G., M.H., and P.S. carried out the statistical analysis and interpretation of the results. A.G. and P.S. obtained the ethical approval for the study; S.G. drew the setup schematic; A.G. wrote the manuscript. All authors discussed the results and commented on the manuscript.

## Conflicts of Interest

The authors declare no conflicts of interest.

## Study Registration

The study was registered as a clinical trial on kofam.ch, the Federal Office of Public Health's (FOPH) portal for human research in Switzerland (trial number SNCTP000004458).

## Previous Presentations

Parts of the study design and pilot data have been presented at the ChiroSuisse & SPS Joint Annual Meeting 2021 in Lausanne, Switzerland (poster presentation).

## Data Availability

https://zenodo.org/record/7811904.
